# Supplementary material for: Rhizosphere Microbe Affects Soil Available Nitrogen and Its Implication for the Ecological Adaptability and Rapid Growth of Dendrocalamus sinicus, the Strongest Bamboo in the World
Source: Int J Mol Sci. 2023 Sep 28;24(19):14665. doi: 10.3390/ijms241914665 (PMC10572720; doi:10.3390/ijms241914665)
Supplement: Supplementary file 1 [file ijms-24-14665-s001.zip › Figure S1.pdf]

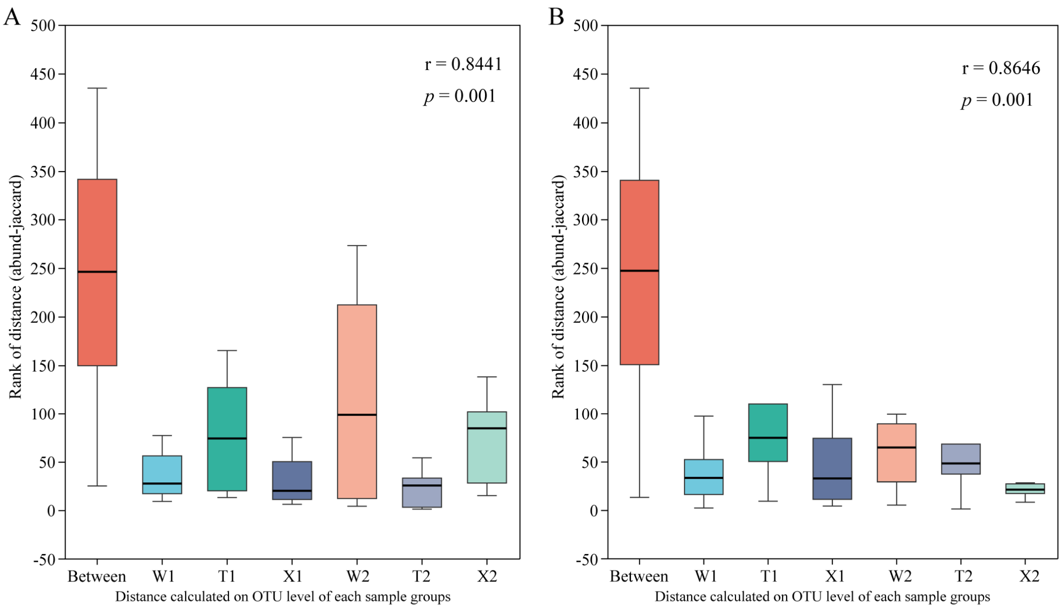

Figure S1. ANOSIM analysis based on abund-jaccard distance of the soil bacterial (A) and fungal (B) communities.

Note: W1, rhizosphere soil sample of bending type in May; W2, rhizosphere soil sample of bending type in August; T1, rhizosphere soil sample of straight type in May; T2, rhizosphere soil sample of straight type in August; X1, rhizosphere soil sample of introduced straight type in May; X2, rhizosphere soil sample of introduced straight type in August.
