# Supplementary material for: Rhizosphere Microbe Affects Soil Available Nitrogen and Its Implication for the Ecological Adaptability and Rapid Growth of Dendrocalamus sinicus, the Strongest Bamboo in the World
Source: Int J Mol Sci. 2023 Sep 28;24(19):14665. doi: 10.3390/ijms241914665 (PMC10572720; doi:10.3390/ijms241914665)
Supplement: Supplementary file 1 [file ijms-24-14665-s001.zip › Figure S2.pdf]

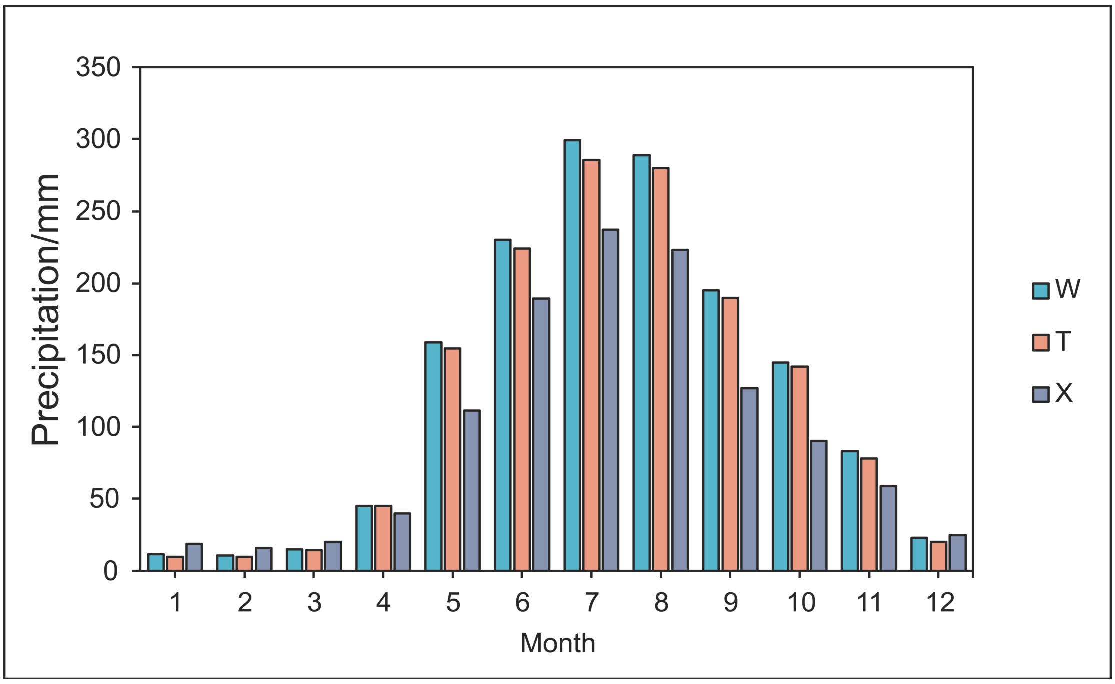

Figure S2. Annual precipitation change.

Note: W, the habitat of bending type; T, the habitat of straight type; X, the habitat of introduced straight type.
