# Supplementary material for: Rhizosphere Microbe Affects Soil Available Nitrogen and Its Implication for the Ecological Adaptability and Rapid Growth of Dendrocalamus sinicus, the Strongest Bamboo in the World
Source: Int J Mol Sci. 2023 Sep 28;24(19):14665. doi: 10.3390/ijms241914665 (PMC10572720; doi:10.3390/ijms241914665)
Supplement: Supplementary file 1 [file ijms-24-14665-s001.zip › Supplementary Tables.pdf]

Table S1. Relative abundance of microbial phylum in soil samples

	Samples	W1/%	T1/%	X1/%	W2/%	T2/%	X2/%
Bacteria	Actinobacteria	40.36	22.99	31.35	44.19	34.99	40.89
	Proteobacteria	32.93	30.05	21.32	29.31	24.69	15.47
	Acidobacteria	10.12	10.62	8.43	6.87	9.55	10.19
	Firmicutes	4.37	9.96	5.04	7.85	14.05	12.25
	Chloroflexi	0.93	6.90	16.99	2.26	3.71	11.40
	Myxococcota	3.33	8.21	5.03	3.63	5.23	3.78
	Verrucomicrobia	1.72	8.31	7.73	1.97	4.77	2.92
	Gemmatimonadetes	0.84	0.92	1.91	1.70	1.24	1.36
	Bacteroidetes	2.09	0.66	0.67	0.58	0.31	0.47
	Planctomycetes	0.45	0.69	0.49	0.61	1.24	0.56
	Nitrospirae	1.88	0.28	0.34	0.57	0.01	0.12
	Samples	W1/%	T1/%	X1/%	W2/%	T2/%	X2/%
Fungi	Ascomycota	68.57	43.28	61.16	47.36	53.56	50.21
	Basidiomycota	23.42	52.09	35.27	47.08	37.83	45.75
	Mortierellomycota	5.73	2.49	0.92	2.77	6.27	1.41
	Glomeromycota	1.99	0.53	1.69	0.46	0.26	1.66
	Kickxellomycota	0.10	0.44	0.07	1.65	1.68	0.04

Note: W1, rhizosphere soil sample of bending type in May; W2, rhizosphere soil sample of bending type in August; T1, rhizosphere soil sample of straight type in May; T2, rhizosphere soil sample of straight type in August; X1, rhizosphere soil sample of introduced straight type in May; X2, rhizosphere soil sample of introduced straight type in August.

Table S2. Analysis of differences in main microbial genera among soil samples

		W1	T1	X1	W2	T2	X2
Bacteria	*Bacillus*	178.8±39.26 c	560.6+53.92 ab	204.4±48.81 c	322.2±86.28 bc	803.0±181.50 a	577.6±131.31 ab
	Bradyrhizobium	279.4±12.05 c	691.8±34.76 a	454.6±66.68 b	665.0±60.95 a	737.8±18.92 a	324.0±24.12 c
	Mycobacterium	104.0±17.99 c	122.0±18.06 bc	213.2±37.27 ab	228.4±51.97 a	193.2±19.23 abc	197.4±32.73 abc
	Kitasatospora	178.4±10.82 bc	132.2±30.21 c	120.0±10.07 c	327.0±17.50 a	277.8±79.15 ab	237.2±37.97 abc
	Sphingomonas	240.8±38.22 ab	146.4±21.56b	308.4±71.09 a	126.2±18.75b	130.0±36.60b	214.4±18.10 ab
	RB41	486.2±49.86 a	300.4±56.21 b	186.2±53.96 b	210.6±69.40 b	230.6±83.25 b	233.4±39.66 b
	Gaiella	453.6±15.32 a	198.6±90.19 bc	212.6±48.42 bc	357.8±75.52 ab	143.2±36.22 c	241.8±46.58 bc
	Haliangium	162.0±17.46 ab	195.4±26.66 a	114.8±17.84 b	133.0±9.71 b	162.6±10.45 ab	128.6±10.16 b
		W1	T1	X1	W2	T2	X2
Fungi	*Cladophialophora*	278.8±70.54 ab	117.0±24.76 ab	491.4±112.76 ab	149.4±52.66 b	232.8±44.55 a	334.6±111.77 ab
	Trichoderma	599.2±119.50 ab	666.2±239.99 ab	306.6±45.35 b	520.4±220.65 ab	877.6±115.64 a	344.2±138.47 b
	Penicillium	307.8±132.28 ab	497.6±70.26 ab	247.0±70.18 ab	198.2±55.15 b	651.4±272.98 a	295.0±58.60 ab
	Apiotrichum	776.6±331.59 a	12.4±6.87 b	1.3±1.18 b	155.0±106.93 b	42.6±23.40 b	1.7±1.35 b
	Fusarium	390.2±101.87 a	27.8±7.08 c	171.0±42.73 bc	53.2±16.73 c	94.4±39.97 bc	230.2±44.66 b
	Exophiala	234.6±76.41 a	62.0±12.21 b	47.0±11.14 b	140.2±71.47 ab	162.8±49.16 ab	16.0±12.50 b
	Acremonium	204.4±33.82 a	48.4±16.76 bc	35.4±6.85 c	106.0±15.11 b	76.6±22.51 bc	67.0±23.87 bc
	Cordana	177.2±76.60 a	5.82±2.28 b	8.22±4.15 b	252.8±77.48 a	24.2±12.96 b	3.04±1.66 b
	Neocosmospora	199.2±29.57 a	2.42±1.02 c	100.2±26.22 b	22.8±9.90 c	1.02±0.30 c	105.4±34.68 b
	Tremellodendropsis	0.1±0.00 b	575.4±421.57 a	0.1±0.00 b	0.28±0.18 b	105.42±67.38 ab	0.1±0.00 b
	Archaeorhizomyces	0.1±0.00 b	382.0±120.49 a	37.82±26.52 b	2.82±1.23 b	320.0±147.58 a	5.46±4.25 b
	Camarophyllus	0.1±0.00 b	617.46±394.69 a	1.06±0.61 b	0.1±0.00 b	0.1±0.00 b	0.1±0.00 b
	Leohumicola	0.1±0.00 b	393.4±149.62 a	39.62±11.04 b	100.2±56.84 b	60.8±18.77 b	0.46±0.22 b

Note: W1, rhizosphere soil sample of bending type in May; W2, rhizosphere soil sample of bending type in August; T1, rhizosphere soil sample of straight type in May; T2, rhizosphere soil sample of straight type in August; X1, rhizosphere soil sample of introduced straight type in May; X2, rhizosphere soil sample of introduced straight type in August. Different letters in the same line indicate significant differences ($p < 0.05$).

Table S3. Analysis of differences in some bacterial functions among soil samples

	W1	T1	X1	W2	T2	X2
chemoheterotrophy	3319.8±105.53 b	2649.8±75.37 c	2905.4±247.40 bc	3922.2±128.44 a	3280.4±255.51 b	3025.4±159.04 bc
aerobic_chemoheterotrophy	3233.4±101.86 b	2618.4±76.90 c	2872.8±246.90 bc	3849.2±119.35 a	3249.4±257.56 b	2985.2±157.73 bc
nitrogen_fixation	283.2±12.00 c	695.2±33.92 a	457.8±67.04 b	668.8±59.63 a	740.2±18.82 a	325.4±23.82 c
cellulolysis	66.2±17.79 b	482.0±127.48 a	634.8±74.05 a	565.6±184.83 a	660.0±133.57 a	422.6±45.80 a
nitrate_reduction	394.2±28.72 a	128.0±25.82 c	131.4±12.14 c	255.0±39.59 b	178.8±17.30 bc	197.4±40.84 bc
ureolysis	73.4±4.69 b	59.2±7.98 b	65.0±5.62 b	65.8±19.38 b	67.6±7.03 b	144.8±44.62 a

Note: W1, rhizosphere soil sample of bending type in May; W2, rhizosphere soil sample of bending type in August; T1, rhizosphere soil sample of straight type in May; T2, rhizosphere soil sample of straight type in August; X1, rhizosphere soil sample of introduced straight type in May; X2, rhizosphere soil sample of introduced straight type in August. Different letters in the same line indicate significant differences ($p < 0.05$).

Table S4. Relative abundance of different functional fungal groups

	Guild	W1/%	T1/%	X1/%	W2/%	T2/%	X2/%
	Dung Saprotroph	2.59	0.12	5.75	0.09	0.03	0.37
	Leaf Saprotroph	0.13	0.02	0.05	0.18	0.03	0.05
	Plant Saprotroph	0.79	0.08	1.36	0.11	0.18	1.47
Saprotrophic	Saprotrophic	0.00	0.00	0.02	0.00	0.00	0.00
	Soil Saprotroph	14.48	6.21	0.74	3.51	6.72	0.36
	Undefined Saprotroph	58.45	84.48	71.80	67.80	71.09	76.71
	Wood Saprotroph	5.45	2.76	2.52	13.87	2.88	5.89
	Sum	81.90	93.67	82.24	85.57	80.93	84.84
	Guild	W1/%	T1/%	X1/%	W2/%	T2/%	X2/%
	Animal Pathogen	6.36	1.99	3.16	8.38	1.73	7.30
	Plant Pathogen	3.17	2.97	5.83	1.44	1.47	3.40
Pathogen	Fungal Parasite	2.93	0.14	0.56	2.16	1.19	0.74
	Lichen Parasite	0.00	0.01	0.00	0.00	0.00	0.01
	Sum	12.46	5.11	9.55	11.98	4.39	11.44
	Guild	W1/%	T1/%	X1/%	W2/%	T2/%	X2/%
	Animal Endosymbiont	0.00	0.00	0.00	0.00	0.00	0.02
	Arbuscular Mycorrhizal	2.93	0.69	2.39	0.86	0.40	2.55
	Ectomycorrhizal	0.34	0.06	0.36	0.39	11.94	0.14
Symbiotroph	Endophyte	0.29	0.10	4.06	1.04	0.36	0.88
	Lichenized	2.08	0.36	1.40	0.16	1.18	0.13
	Orchid Mycorrhizal	0.00	0.01	0.00	0.00	0.81	0.00
	Sum	5.64	1.22	8.21	2.44	14.69	3.72

Note: W1, rhizosphere soil sample of bending type in May; W2, rhizosphere soil sample of bending type in August; T1, rhizosphere soil sample of straight type in May; T2, rhizosphere soil sample of straight type in August; X1, rhizosphere soil sample of introduced straight type in May; X2, rhizosphere soil sample of introduced straight type in August. Different letters in the same line indicate significant differences ($p < 0.05$).

Table S5. Soil microbial communities with significant differences

	Species name	group	Mean	LDA_value	Pvalue
Bacteria	*Pedomicrobium*	W1	4.767345	4.433512	0.000579
	Solirubrobacter	W1	4.687853	4.405673	0.000112
	Gaiella	W1	5.023153	4.621723	0.007118
	Micromonospora	W1	4.481648	4.164797	0.004619
	Arthrobacter	W1	4.527378	4.231651	0.002643
	Nocardioides	W1	4.780194	4.442551	0.000762
	Reyranella	W1	4.354520	4.155697	0.000342
	RB41	W1	5.050913	4.607000	0.022805
	MND1	W1	4.682856	4.424279	0.000602
	Haliangium	W1	4.574262	4.020340	0.030672
	Kitasatospora	W2	4.777100	4.286802	0.006644
	Streptomyces	W2	4.774355	4.383982	0.016476
	Luedemannella	W2	4.663654	4.295329	0.000374
	Candidatus_Solibacter	T1	4.689228	4.236479	0.013984
	Anaeromyxobacter	T1	4.749679	4.408057	0.000350
	Bacillus	T2	5.134566	4.689938	0.007352
	Bradyrhizobium	T2	5.097985	4.523772	0.000654
	Candidatus_Udaeobacter	X1	4.987426	4.565805	0.012744
	Acidothermus	X1	5.072493	4.749311	0.011610
	Sphingomonas	X1	4.759445	4.293756	0.005530
	HSB_OF53-F07	X1	4.761487	4.419299	0.020989
	Bryobacter	X2	4.515087	4.105184	0.001488
	Actinoallomurus	X2	4.420967	4.148947	0.026955
	Pseudonocardia	X2	4.376521	4.064796	0.000294
	Conexibacter	X2	4.766628	4.370470	0.003992
	FCPS473	X2	4.876113	4.561331	0.000403
	Species name	group	Mean	LDA_value	Pvalue
Fungi	*Gonytrichum*	W1	4.486678	4.303970	0.009794
	Acremonium	W1	4.650954	4.478092	0.009031
	Neocosmospora	W1	4.627346	4.478850	0.000169
	Mortierella	W1	4.994712	4.649244	0.030236
	Apiotrichum	W1	5.198802	4.886796	0.000625
	Exophiala	W1	4.713527	4.482962	0.005590
	Fusarium	W1	4.914217	4.566031	0.000530
	Saitozyma	W2	5.205070	4.836008	0.016033
	Trechispora	W2	5.037194	4.684383	0.016924
	Cordana	W2	4.666829	4.458914	0.001406
	Hygrocybe	W2	4.731303	4.435804	0.012853
	Metarhizium	W2	4.857473	4.494690	0.028028
	Leohumicola	T1	4.827901	4.510476	0.000859
	Archaeorhizomyces	T1	4.765919	4.576460	0.000825
	Pseudallescheria	T2	4.847206	4.694836	0.044360

Cladophialophora	X1	5.075464	4.736573	0.015213
Entoloma	X1	4.902233	4.647627	0.006644
Agaricus	X1	5.189211	4.852097	0.010651
Clavaria	X2	5.492799	5.165414	0.006982
Chaetomium	X2	4.996272	4.708027	0.002541

Note: W1, rhizosphere soil sample of bending type in May; W2, rhizosphere soil sample of bending type in August; T1, rhizosphere soil sample of straight type in May; T2, rhizosphere soil sample of straight type in August; X1, rhizosphere soil sample of introduced straight type in May; X2, rhizosphere soil sample of introduced straight type in August.

Table S6. RDA analysis results of microbial (bacterial, fungal) communities

		RDA1	RDA2	r^2	*p* values
Bacteria	SWC	-0.4303	0.9027	0.5924	0.001
	pH	0.7565	0.654	0.7109	0.001
	SOC	-0.4547	0.8907	0.7487	0.001
	AP	-0.8914	-0.4532	0.4533	0.001
	AK	0.0756	0.9971	0.6416	0.001
	NH_4^+-N	-0.1984	0.9801	0.6989	0.001
	C: N	-0.1186	-0.9929	0.0452	0.524
	NH_4^+: NO_3^-	-0.9512	0.3085	0.3666	0.001
		CCA1	CCA2	r^2	*p* values
Fungi	SWC	-0.9517	-0.3069	0.5736	0.001
	pH	-0.0908	-0.9959	0.7727	0.001
	SOC	-0.997	-0.0778	0.8020	0.001
	AP	-0.3842	0.9233	0.7794	0.001
	AK	-0.7637	-0.6455	0.8171	0.001
	$NH4^+$-N	-0.8827	-0.4699	0.5437	0.001
	C: N	-0.4829	0.8757	0.0298	0.668
	NH_4^+: NO_3^-	-0.7987	0.6017	0.2737	0.010

Note: SWC, soil water content; SOC, soil organic carbon; AP, available phosphorus; AK, available potassium; NH_4^+-N, ammonia nitrogen: C: N, the ratio between soil organic carbon and soil total nitrogen; NH_4^+: NO_3^-, the ratio between ammonia nitrogen and nitrate nitrogen.
